# Supplementary material for: Hyperoxia induces glutamine-fuelled anaplerosis in retinal Müller cells
Source: Nat Commun. 2020 Mar 9;11:1277. doi: 10.1038/s41467-020-15066-6 (PMC7062830; doi:10.1038/s41467-020-15066-6)
Supplement: Supplementary file 1 — Supplementary Information [file 41467_2020_15066_MOESM1_ESM.pdf]

Hyperoxia induces glutamine-fueled anaplerosis in retinal Müller cells

Charandeep Singh[1], Vincent Tran[1], Leah McCollum[1], Youstina Bolok[1], Kristin Allan[1,2], Alex Yuan[1], George Hoppe[1], Henri Brunengraber[3], and Jonathan E. Sears[1,4]

[1]Ophthalmic Research, Cole Eye Institute, Cleveland Clinic Foundation, Cleveland, OH 44195

[2]Molecular Medicine, Case Western Reserve School of Medicine Cleveland, OH 44106

[3]Department of Nutrition, Case Western Reserve School of Medicine Cleveland, OH 44106

[4]Cardiovascular and Metabolic Sciences, Cleveland Clinic, Cleveland, OH 44195

Corresponding author: Jonathan E. Sears Tel: 216-2444-8157; Email: searsj@ccf.org

Supplementary Figures and Legends

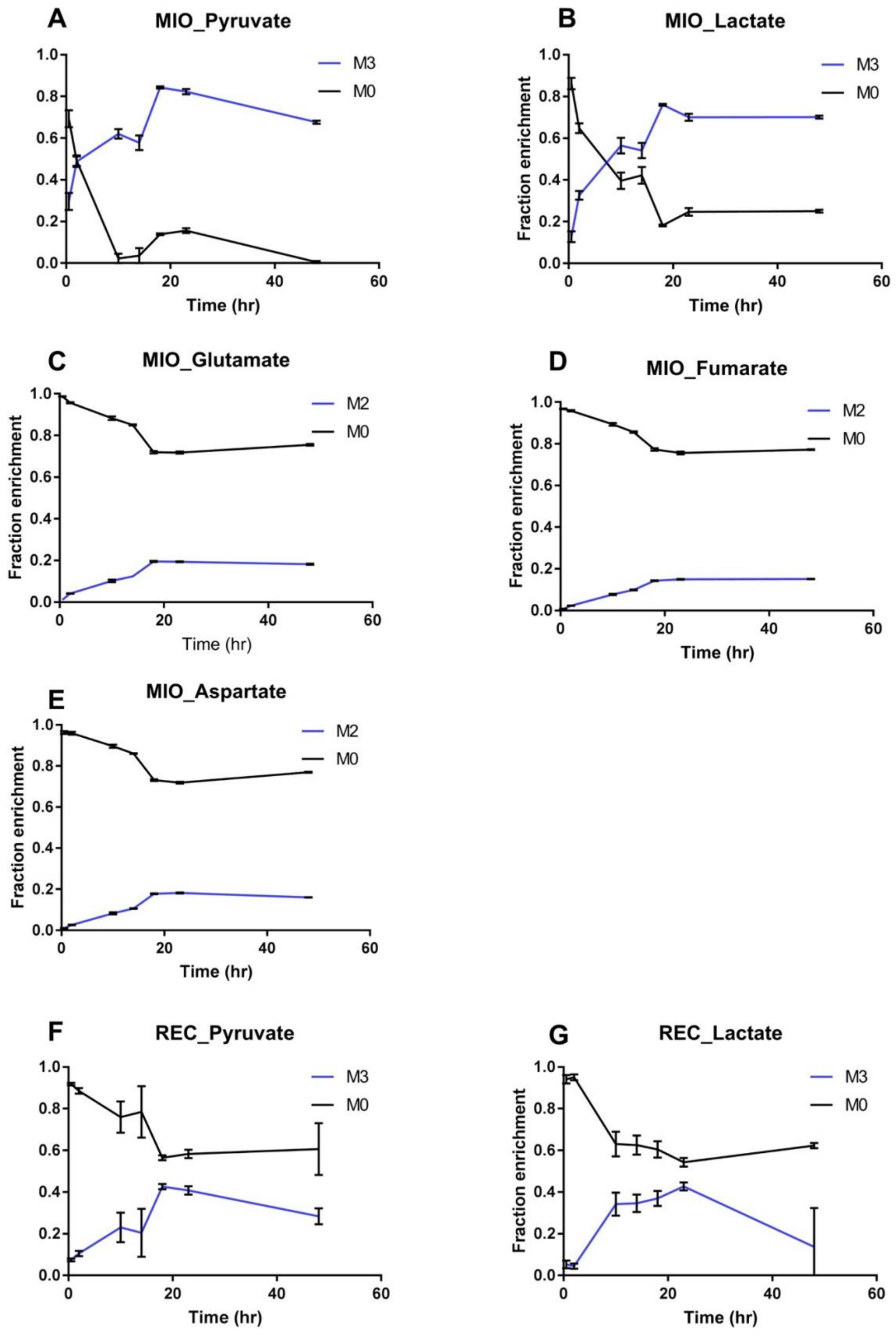

Supplementary Figure 1. Isotopic steady state. To find out how long it takes to reach isotopic steady state in MIO-M1 and REC cells, the cells lines were maintained in $[^{13}C_6]$ glucose media and intracellular metabolites were extracted from the cells at given time points. A-E) MIO-M1 cells (n=3 per condition per time point, mean +/- SEM). F-G) Retinal endothelial cells (n=3 per condition per time point, mean +/- SEM).

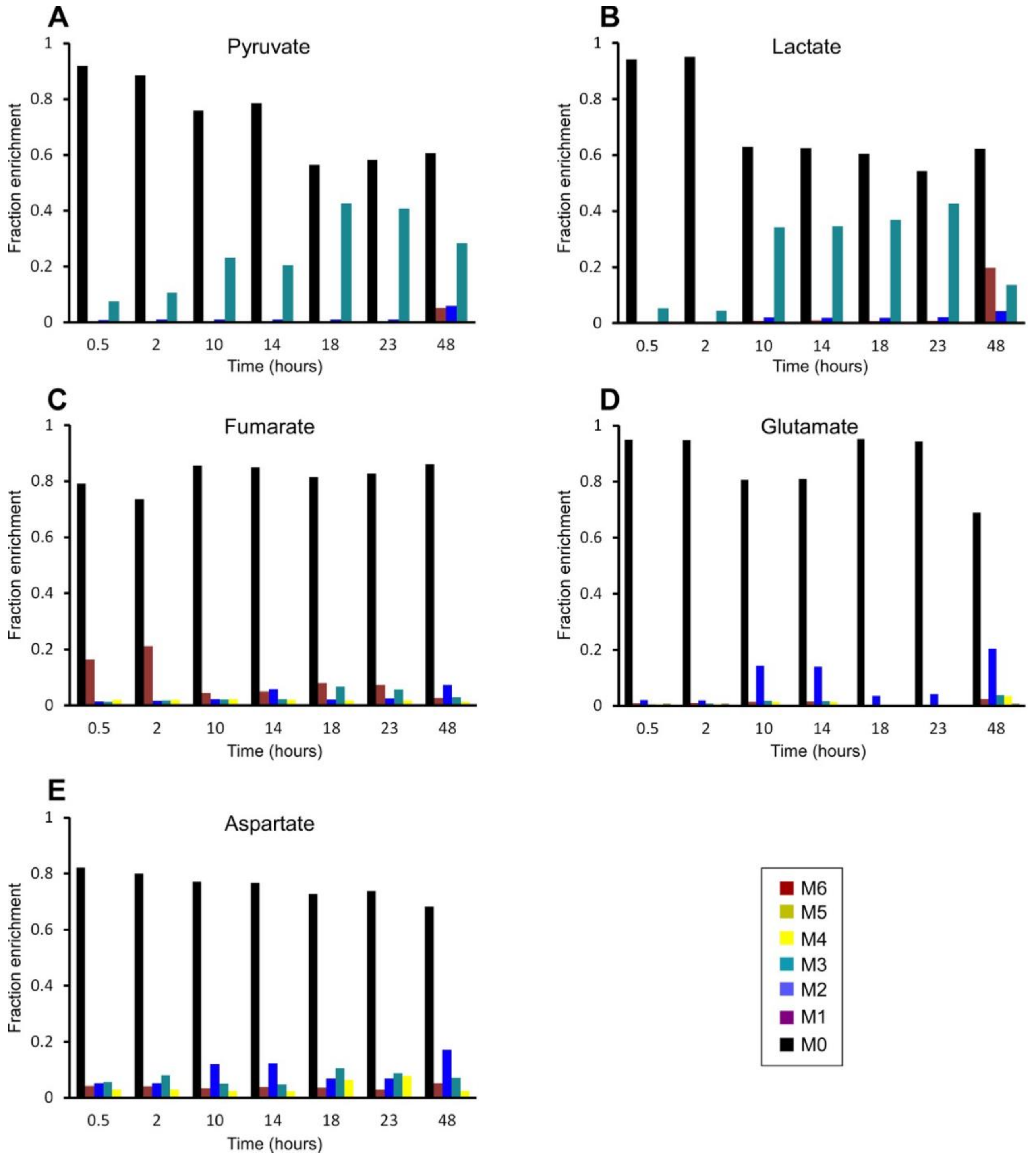

Supplementary Figure 2. Retinal endothelial cells have low TCAC rate. MIDs for glycolytic and TCAC metabolites A) Pyruvate, B) Lactate, C) Fumarate, D) Glutamate, E) Aspartate clearly show very slow labeling of TCAC metabolites as compared to glycolytic metabolites in retinal endothelial cells, implying low flux is diverted to TCAC from glycolytic end products (n=3 biological independent samples per time point per condition).

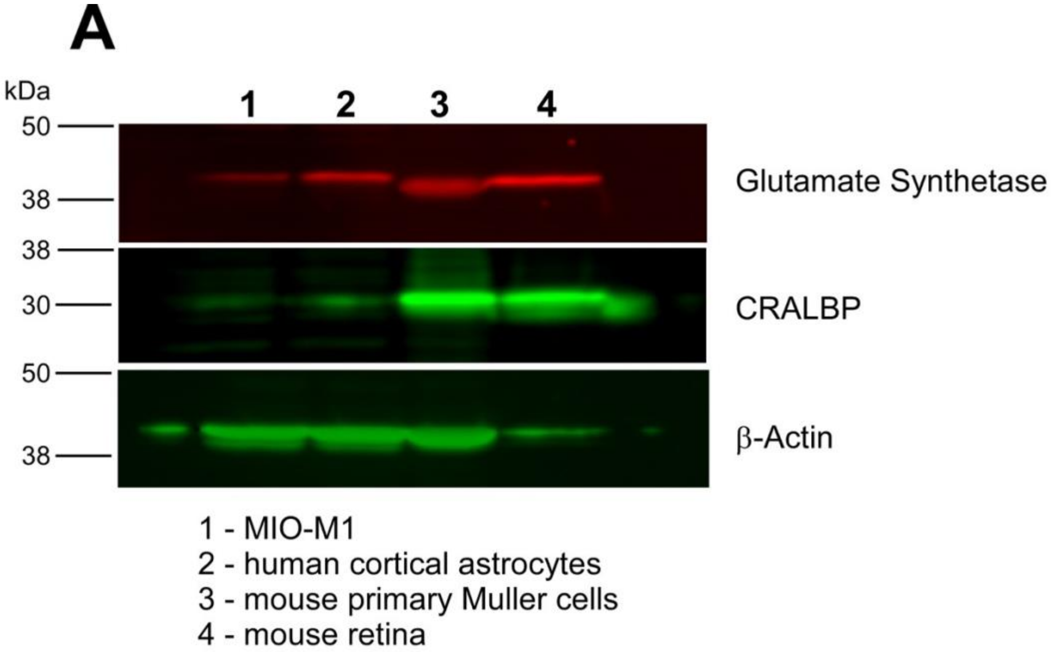

B

	**CRALBP**, RFI/μg	**GS**, RFI/μg	**CRALBP/Act**, RFI ratio	**GS/Act**, RFI ratio	**CRALBP/GS**, RFI ratio	**GS/CRALBP**, RFI ratio
**MIO-M1**	30	6	0.02	0.004	0.206	4.9
**Astrocytes**	103	23	0.07	0.014	0.219	4.6
**Muller cells**	1206	29	0.83	0.020	0.024	42.2
**Retina**	6303	345	4.65	0.254	0.055	18.3

Supplementary Figure 3. Levels of glial markers in cultures of glial cells used in the study in comparison to that of the retina. Protein extracts of cultured MIO-M1 cells (1), primary human cortical astrocytes (2), primary mouse retinal Müller cells, and whole mouse retina (4) were separated on 4%-20% PAGE, electrotransferred to PVDF and probed with the mixture of mouse monoclonal antibody against glutamine synthetase (GS) (BD Bioscience, dilution 1:1,000) and rabbit polyclonal antibody UW55 against cellular retinaldehyde binding protein (CRALBP) originally raised by Jack Saari (University of Washington) and gifted to us by John W. Crabb (Cleveland Clinic). Bound primary antibody were revealed by secondary anti-mouse and anti-rabbit antibodies conjugated to IRDye 680RD and IRDye 800CW far-red fluorescent dyes, respectively (LiCor, Lincoln, NE, dilution 1:10,000). PVDF membranes were then stripped in Restore PLUS buffer (ThermoFisher), blocked again and re-probed with rabbit monoclonal anti β-actin antibody (Cell Signaling Technology, 1:1,000) followed by anti-rabbit-IRDye 800CW secondary antibody. Imaging and quantitative densitometry were conducted using LiCor scanner and LiCor Image Suite v5.2.5 software. A) Representative images of GS (52 kDa band) and CRALBP (36 kDa band, two independent isolations of mouse primary retinal Müller cell were performed and two western botting analyses were run, respectively) and (B) relative abundance of GS and CRALBP in protein extracts expressed as relative fluorescence intensity RFI normalized to total protein loaded, which were 28 µg/ lane (1-3 in Figure S3A) or 5 µg/lane (4), or to RFI of β-actin.

Figure S4

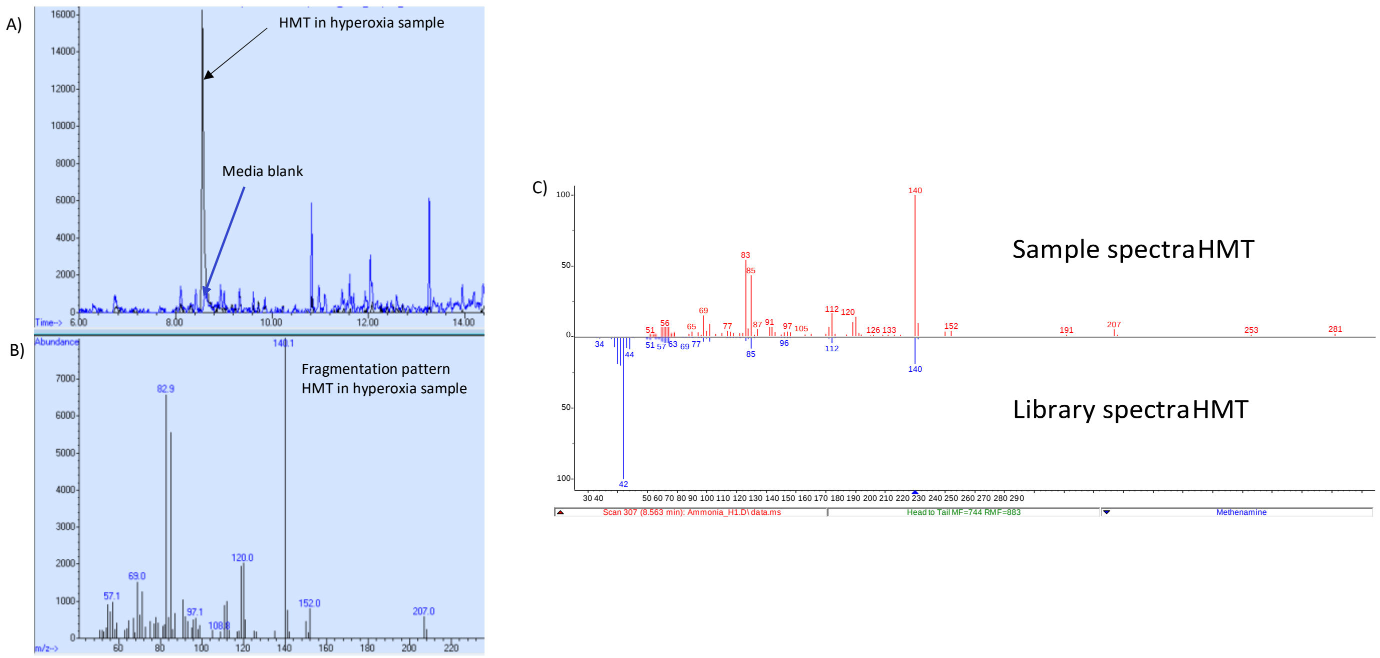

Supplementary Figure 4. HMT detected in hyperoxic MIO-M1 cells media. Media sample was taken from cells cultured in normoxic vs. hyperoxic incubator after 48 h. Ammonium was converted to HMT by reacting with formaldehyde and measured on GC-MS. A) Overlaid extracted ion chromatogram for m/z 140 corresponding to hexamethylenetetramine in spent media vs. unspent media. HMT peak was only detected in spent media, both in normoxic and hyperoxic samples, but not in unspent media. B) Fragmentation pattern of HMT peak detected in the spent media. C) Comparison of HMT spectra in the sample with the HMT spectra in the NIST2017 library.

Figure S5

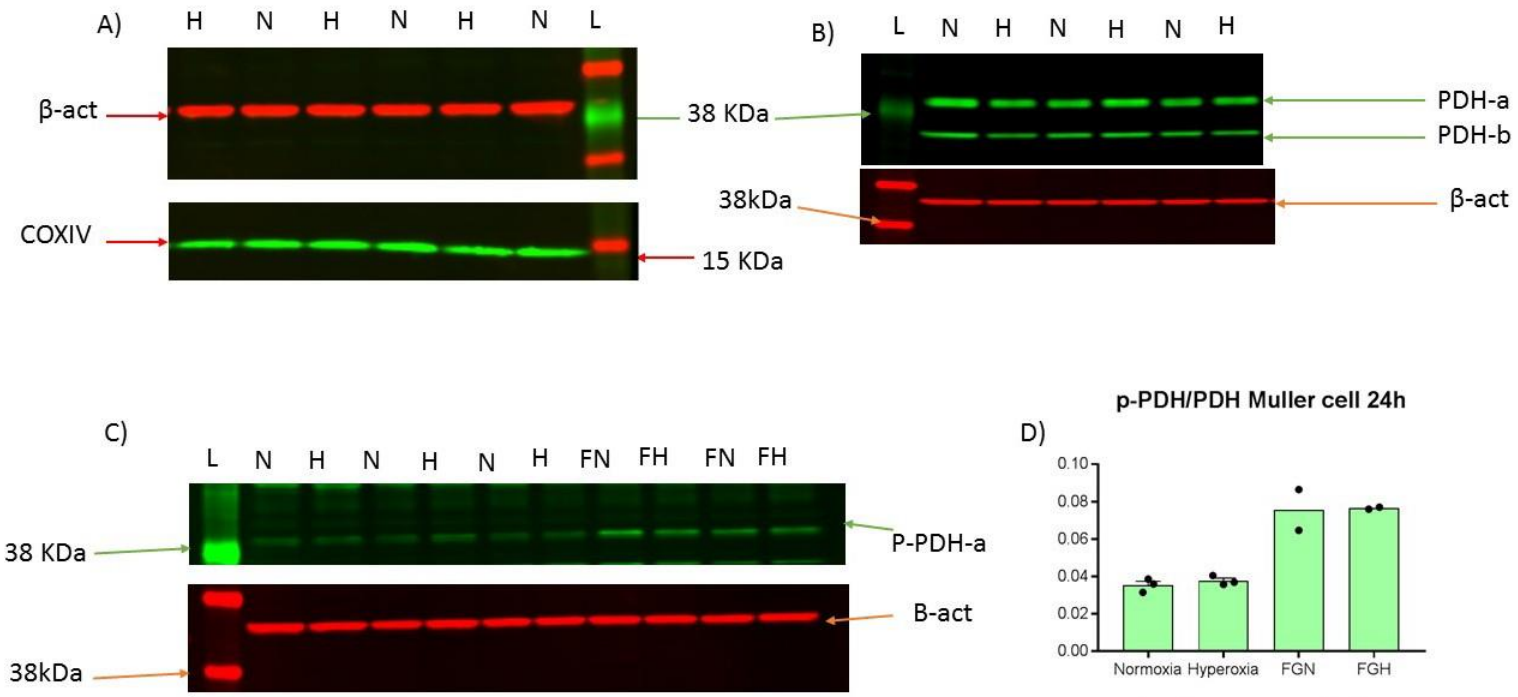

- note: these are only two replicates in each FG condition

Supplementary Figure 5. Mitochondrial loss or PDK dependent phosphorylation of PDH (p-Ser232) is not responsible for reduced MIO-M1 cells pyruvate to TCAC flux and subsequent loss of glutamine production by MIO-M1 cells. Western blot showing mitochondrial marker COXIV, pyruvate dehydrogenase PDH, phosphorylated pyruvate dehydrogenase p-PDH. Proteins extracted from MIO-M1 cells incubated in hyperoxia for 24 h were compared with those placed in normoxic condition. A) There was no difference in COXIV levels between normoxic vs. hyperoxic condition, implying that the number of mitochondria were unchanged. B) No difference in PDH levels was seen between normoxic vs. hyperoxic condition. C) Next we tested difference in phosphorylated PDH in normoxic vs. hyperoxic conditions, and found no difference between hyperoxic conditions. We used HIF stabilized cells as control, and found clear increased concentration of p-PDH levels. Three biological replicates. D) Ratio of phosphorylated PDH/ total PDH in normoxia vs. hyperoxia incubated MIO-M1 cells, with and without HIF stabilizer FG-4592. H, hyperoxia; N, normoxia; L, Molecular ladder; MW, Molecular weight in kDa, FGN, FG-4592 treated normoxic cells; FGH, FG-4592 treated hyperoxic cells (n=3 for normoxic and hyperoxic samples; n=2 for FGN and FGH samples).

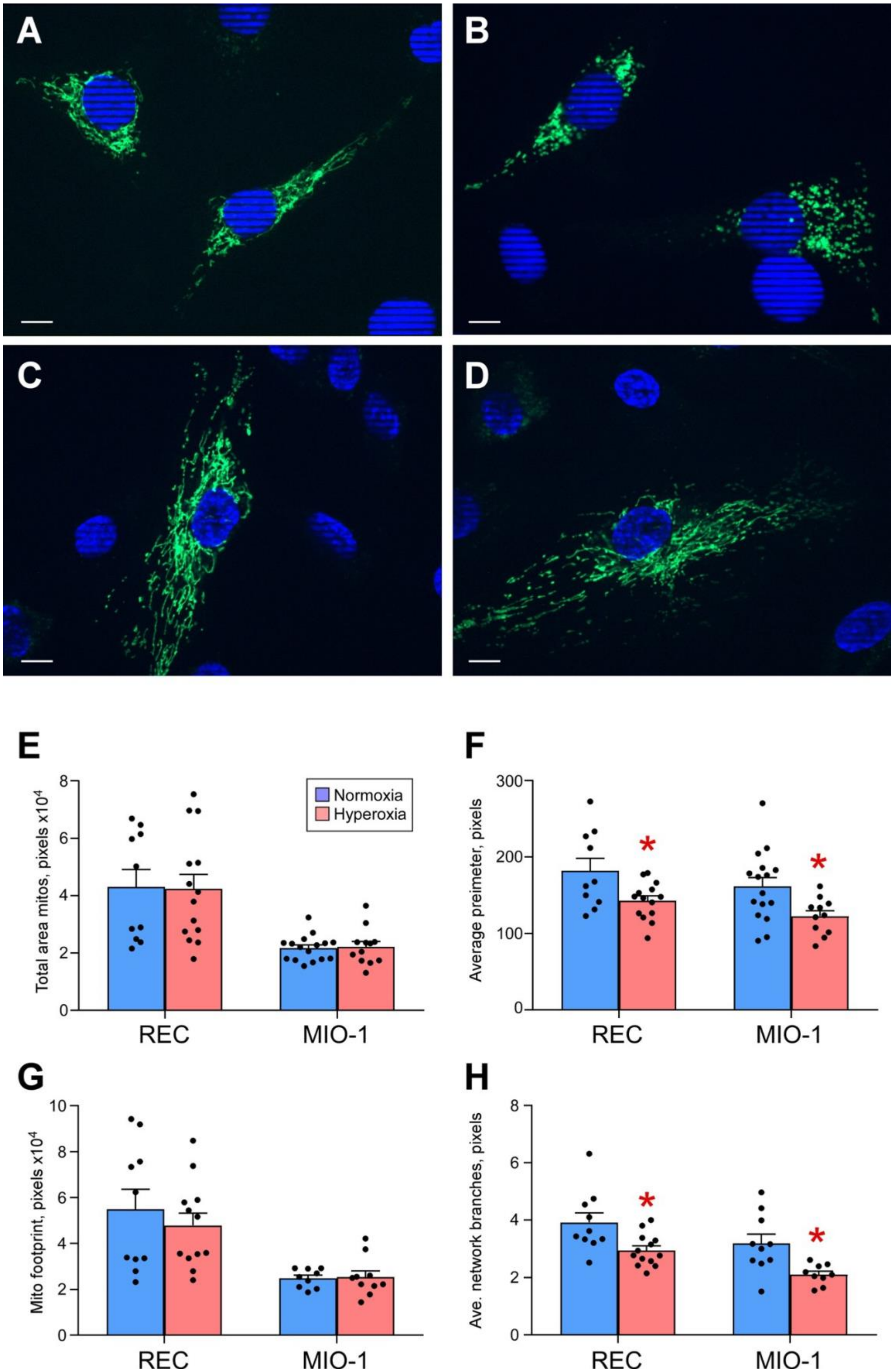

Supplementary Figure 6. Mitochondrial staining of MIO-M1 cells and RECs in normoxia versus hyperoxia. Cells were transfected with E1α pyruvate dehydrogenase fused to emerald GFP. A) Normoxic MIO-M1 cells demonstrate string-like morphology of mitochondria. B) MIO-M1 cells in hyperoxia demonstrate mitochondria clumping and are devoid of interconnecting string-like pattern. RECs in normoxia (C) and hyperoxia (D) demonstrate similar extensive mitochondrial network without evident effect of hyperoxia. E) Total area of mitochondria per individual cell and (F) average mitochondrion perimeter as calculated by Mitophagy ImageJ macro indicates that hyperoxia results in smaller size, fragmented mitochondrial network, *REC normoxia vs

hyperoxia p= 0.019, MIO-M1 normoxia vs hyperoxia p= 0.017. G) Mitochondrial "footprint" (essentially area occupied by mitochondria) and (H) the average number of each individual mitochondrial network branches measured by MiNA ImageJ macro demonstrates hypoxia-induced reduction in branched networks, i.e., involution of the mitochondrial tree, *REC normoxia vs hyperoxia p= 0.01, MIO-M1 normoxia vs hyperoxia p= 0.008. (REC Normoxia n=10, REC Hyperoxia n=14, MIO-M1 Normoxia n=16, MIO-M1 Hyperoxia n=12 individual cells analysed). Mitochondrial staining experiments were independently performed twice with BacMan 2.0 staining (shown) and twice with MitoTracker Red (not shown) with similar results. Scale bar = 10 µm.

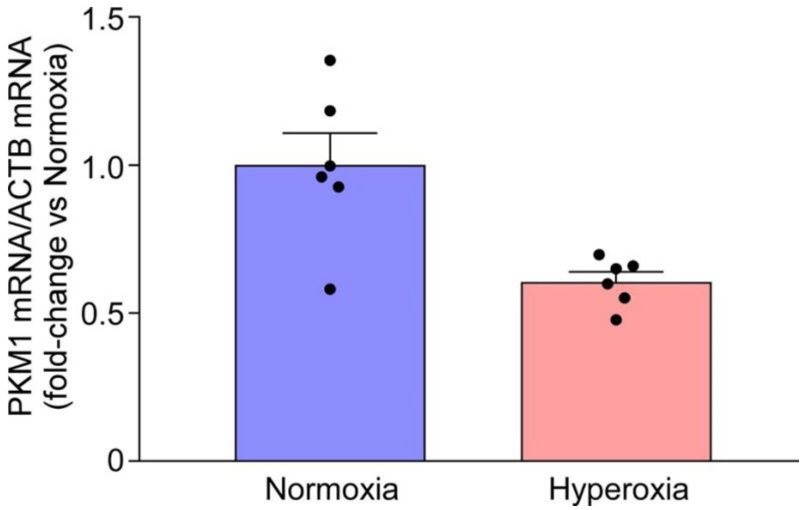

Supplementary Figure 7. PKM1 is expressed in MIO-M1 cells in culture. We measured the expression of PKM1 and 2 in MIO-M1 cells incubated either in normoxic or hyperoxic incubator (n=6 biologically independent samples (cell culture wells) for each condition, mean +/- SEM).
