# Supplementary material for: Hyperoxia induces glutamine-fuelled anaplerosis in retinal Müller cells
Source: Nat Commun. 2020 Mar 9;11:1277. doi: 10.1038/s41467-020-15066-6 (PMC7062830; doi:10.1038/s41467-020-15066-6)
Supplement: Supplementary file 3 — Reporting Summary [file 41467_2020_15066_MOESM3_ESM.pdf]

nature research

Corresponding author(s): Jonathan Sears

Last updated by author(s): 01/30/2020

Reporting Summary

Nature Research wishes to improve the reproducibility of the work that we publish. This form provides structure for consistency and transparency in reporting. For further information on Nature Research policies, see Authors & Referees and the Editorial Policy Checklist.

Statistics

For all statistical analyses, confirm that the following items are present in the figure legend, table legend, main text, or Methods section.

n/a	Confirmed	
☐	☒	The exact sample size (*n*) for each experimental group/condition, given as a discrete number and unit of measurement
☐	☒	A statement on whether measurements were taken from distinct samples or whether the same sample was measured repeatedly
☐	☒	The statistical test(s) used AND whether they are one- or two-sided<br>*Only common tests should be described solely by name; describe more complex techniques in the Methods section.*
☒	☐	A description of all covariates tested
☒	☐	A description of any assumptions or corrections, such as tests of normality and adjustment for multiple comparisons
☐	☒	A full description of the statistical parameters including central tendency (e.g. means) or other basic estimates (e.g. regression coefficient) AND variation (e.g. standard deviation) or associated estimates of uncertainty (e.g. confidence intervals)
☐	☒	For null hypothesis testing, the test statistic (e.g. *F*, *t*, *r*) with confidence intervals, effect sizes, degrees of freedom and *P* value noted<br>*Give P values as exact values whenever suitable.*
☒	☐	For Bayesian analysis, information on the choice of priors and Markov chain Monte Carlo settings
☒	☐	For hierarchical and complex designs, identification of the appropriate level for tests and full reporting of outcomes
☒	☐	Estimates of effect sizes (e.g. Cohen's *d*, Pearson's *r*), indicating how they were calculated

Our web collection on statistics for biologists contains articles on many of the points above.

Software and code

Policy information about availability of computer code

Data collection

Metabolomics: Agilent GC-MS + software (Mass Hunter with MSD ChemStation and Metabolite detector)
Western Blot: Li-Cor Odyssey CLx Image Studio 5.2
qPCR: ABI7900HT SDS 2.4
Immunofluorescent microscopy: Zeiss Imager.Z1 ZEN 2.5pro
PerkinElmer 2030 Workstation for Victor X2 plate fluorimeter
SoftMax Pro 5 for SpectraMax PLUS-384 plate spectrophotometer

Data analysis

Metabolomics: Mass hunter, Metabolite detector, Graphpad prism 7 and Microsoft Excel 2011
Western blot densitometry: Image Studio 5.2
qPCR: Microsoft Excel 2011
Statistical analyses: Microsoft Excel 2011, GraphPad Prizm 7
Isocor version 2 and NIST17 (Agilent) were used
NIH ImageJ v1.40g with two custom macros Mitophagy v1.44 and MiNA (Valente et al, Acta Histochem 119, 315, 2017)

For manuscripts utilizing custom algorithms or software that are central to the research but not yet described in published literature, software must be made available to editors/reviewers. We strongly encourage code deposition in a community repository (e.g. GitHub). See the Nature Research guidelines for submitting code & software for further information.

Data

Policy information about availability of data

All manuscripts must include a data availability statement. This statement should provide the following information, where applicable:

- Accession codes, unique identifiers, or web links for publicly available datasets
- A list of figures that have associated raw data
- A description of any restrictions on data availability

All relevant data related to this manuscript are available on request from the authors on reasonable request. Metabolomics data is available online via metabolights study ID MTBLS1228 Hyperlink: [https://www.ebi.ac.uk/metabolights/MTBLS1228]. It is currently under curation by Metabolights and can take 2-3 weeks.

Field-specific reporting

Please select the one below that is the best fit for your research. If you are not sure, read the appropriate sections before making your selection.

☒ Life sciences ☐ Behavioural & social sciences ☐ Ecological, evolutionary & environmental sciences

For a reference copy of the document with all sections, see nature.com/documents/nr-reporting-summary-flat.pdf

Life sciences study design

All studies must disclose on these points even when the disclosure is negative.

Sample size	No sample-size calculation was performed, but rather determined based on best practices in the corresponding field of research, e.g., n=>6 qPCR, n=>3 for western blotting, etc. Sample size and statistical significance is given for each experiment. Samples size of more than 4 were used in all the conditions for metabolomic analysis. More than 4 samples provides n-1=3 degrees of freedom at least and therefore normality can be assumed while computing measures of dispersion or hypothesis testing. Only for steady-state determination 3 samples were used.
Data exclusions	No data was excluded. ALTERNATIVELY: Outlier data points in mitochondrial analysis were excluded based on outlier analysis using GraphPad Prism 7.05 with build-in ROUT method and Q = 10%.
Replication	4-6 biological replicates were used each time. Only for steady-state labeling 3 samples were used. All experimental repeats gave similar results.
Randomization	Measurements were randomised. Samples were mixed and measured randomly on mass spec.
Blinding	Only partial blinding was possible, i.e., the technical assistants who processed samples were blind to the nature of samples' content, but the leading researchers were aware of the experimental conditions tested.

Reporting for specific materials, systems and methods

We require information from authors about some types of materials, experimental systems and methods used in many studies. Here, indicate whether each material, system or method listed is relevant to your study. If you are not sure if a list item applies to your research, read the appropriate section before selecting a response.

Materials & experimental systems

n/a	Involved in the study	
☐	☒	Antibodies
☐	☒	Eukaryotic cell lines
☒	☐	Palaeontology
☐	☒	Animals and other organisms
☒	☐	Human research participants
☒	☐	Clinical data

Methods

n/a	Involved in the study	
☒	☐	ChIP-seq
☒	☐	Flow cytometry
☒	☐	MRI-based neuroimaging

Antibodies

Antibodies used

Rabbit anti-COXIV (Cat #4844, Lot #3, Cell Signaling Technology, dilution 1:1000)
Rabbit anti-PDH (Cat #ABS2082, Lot #Q2792883, Sigma/Millipore, dilution 1:1000)
Rabbit anti-phospho-PDH (Cat #AP1063, Lot #3086643, Sigma/Millipore, dilution 1:1000)
Mouse anti-beta-actin (Cat #3700, Lot #17, Cell Signaling Technology, dilution 1:2,000)
Rabbit anti-beta-actin (Cat #4970, Lot #15, Cell Signaling Technology, dilution 1:2,000)
Mouse anti-glutamine synthetase (Cat #610517, Lot #8255557, BD Bioscience, dilution 1:1,000)
Rabbit anti-CRALBP (Clone UW55, from Jack Saari, dilution 1:1,000)

	Donkey anti-rabbit IgG 800CW-conjugated (Cat #925-32213, LiCor Biosciences, dilution 1:10,000)<br>Donkey anti-rabbit IgG 680RD-conjugated (Cat #925-68073, LiCor Biosciences, dilution 1:10,000)<br>Donkey anti-mouse IgG 800CW-conjugated (Cat #926-32212, LiCor Biosciences, dilution 1:10,000)<br>Donkey anti-mouse IgG 680RD-conjugated (Cat #926-68072, LiCor Biosciences, dilution 1:10,000)
Validation	All but one commercially available antibodies were validated for western blotting by the corresponding manufacturer and used according to manufacturer recommendations, i.e., dilution, protocol and method of detection. Statements of validation, species specificity, relevant references are readily available at the corresponding manufacturer webpages. Anti-CRALBP UW55 antibody was thoroughly tested and validated by a great number of independent research labs that is also evidenced by more than 200 peer-reviewed publications, e.g., Xue Y, et al. J Clin Invest 2015. PMID 25607845, Collery R, et al. Invest Ophthalmol Vis Sci 2008. PMID 18502992, Dunn KC, et al. Exp Eye Res 1996, Kennedy BN, et al. Exp Eye Res 2003, Akrami H, et al. Biochem Genet 2011, Boppana S, et al. Biochim Biophys Acta 2012.

Eukaryotic cell lines

Policy information about cell lines

Cell line source(s)	Human microvascular retinal endothelial cells were procured from a commercial source - Cell Systems (Kirkland, WA, USA). Müller cells (MIO-M1) were a kind gift from Dr. A. Limb (UCL Institute of Ophthalmology, London, UK). Primary astrocytes were provided by Dr. Jessica Williams through an MTA from ScienCell (LRI, Cleveland Clinic, OH) and primary muller cells were isolated by Kristin Allan (Ophthalmic research, Cleveland Clinic, OH)
Authentication	Neither cell line was independently validated by us.
Mycoplasma contamination	We did not perform analysis for mycoplasma contamination.
Commonly misidentified lines<br>(See ICLAC register)	Commonly misidentified lines were not used for this study.

Animals and other organisms

Policy information about studies involving animals; ARRIVE guidelines recommended for reporting animal research

Laboratory animals	C57BL/6J newborn mouse pups from postnatal day 6 to 17 of both genders equally were used in this study.Animals were housed in their regular cages with unrestricted access to food and water. During experiments newborn pups were kept with their mothers. Following environmental provisions were available: environmental temperature of 68–79°F, relative humidity of 30 –70%, and 14 h:10 h light:dark cycle.
Wild animals	*Provide details on animals observed in or captured in the field; report species, sex and age where possible. Describe how animals were caught and transported and what happened to captive animals after the study (if killed, explain why and describe method; if released, say where and when) OR state that the study did not involve wild animals.*
Field-collected samples	*For laboratory work with field-collected samples, describe all relevant parameters such as housing, maintenance, temperature, photoperiod and end-of-experiment protocol OR state that the study did not involve samples collected from the field.*
Ethics oversight	All treatments of animals (mice) were approved by the Cleveland Clinic Institutional Animal Care and Use Committee (IACUC) under the protocol # 2019-2183.

Note that full information on the approval of the study protocol must also be provided in the manuscript.
